# Designing a sustainable strategy for malaria control?

**DOI:** 10.1186/1475-2875-10-220

**Published:** 2011-08-03

**Authors:** Clive Shiff, Phil Thuma, David Sullivan, Sungano Mharakurwa

**Affiliations:** 1 Department of Molecular Microbiology and Immunology and Johns Hopkins Malaria Research Institute, Johns Hopkins Bloomberg School of Public Health, Baltimore MD 21205 USA; 2 The Malaria Institute at Macha and the Macha Research Trust, P.O. Box 630166, Choma, Zambia

## Abstract

Malaria in the 21^st ^century is showing signs of declining over much of its distribution, including several countries in Africa where previously this was not thought to be feasible. Yet for the most part the strategies to attack the infection are similar to those of the 1950s. Three major Journals have recently drawn attention to the situation, stressing the importance of research, describing the successes and defining semantics related to control. But there is a need to stress the importance of local sustainability, and consider somewhat urgently how individual endemic countries can plan and implement the programmes that are currently financed, for the most part, by donor institutions. On an immediate basis research should be more focused on a data driven approach to control. This will entail new thinking on the role of local infrastructure and in training of local scientists in local universities in epidemiology and field malariology so that expanded control programmes can become operational. Donor agencies should encourage and facilitate development of career opportunities for such personnel so that local expertise is available to contribute appropriately.

## Background

Malaria is one of the most important public health concerns in the early stages of the 21^st ^century and has drawn attention globally at a high scientific and international level. The journals, Science (14^th ^May 2010), Nature (20^th ^May 2010) and the Lancet (6^th ^November 2010) have editorialized on malaria, on basic research advances as well as the success of malaria control interventions in several endemic countries. While progress in the field is certainly impressive, sustainability will require improvements, such as tracking parasite reservoirs, better use of technology in case detection and epidemiology, to continue pushing back on the disease. It is important to remember that some of the key tools that have contributed so much to the success are ephemeral. They must be used efficiently and effectively and much has yet to be done to integrate them into the local public health infrastructures while they are still effective.

The successes achieved in most African countries have been through the massive global effort with mobilization of international and local resources and expanding research on malaria. Undoubtedly, this achievement has happened because of considerable public interest, leadership of international agencies, major funding organizations and national programmes. These have been melded with the support of progressive governments in some endemic countries which have worked to implement programmes and now declines in the prevalence of malaria are seen in a variety of countries and regions including those in the perceived heartland of this lethal parasite, such as in West Africa[1], East Africa [2], and Central Africa [3]. Much of this success has occurred through strategic use of interventions initially laid out and utilized over 50 years ago [4] and now augmented with recent technological advances. These include a highly effective drug, artemisinin and its combinations that have become available for widespread use, and recent methods to apply insecticides on mosquito nets. These treated nets have caught the public attention to such an extent that much of the global public now raise funds and create awareness of the plight of those living in endemic areas. However, public health authorities know full well that the situation is dynamic and dependent on the efficacy of the drugs and insecticides currently in use. In spite of ongoing massive research efforts, the entire intervention globally against malaria is essentially dependent on one family of insecticides that, impregnated into polyester, maintain an effect for several years and one drug class based on artemisinin. Resistance to both these compounds is apparent or suspected and may soon spread. Therefore, time is limited; the strategy is vulnerable and it is time to move to the next phase of the anti-malaria strategy that must involve better local infrastructure and a sustained public health approach.

Attention must be drawn to this vulnerable, potentially unsustainable and ephemeral strategy and research needs to be focused on ameliorating this situation. However, there is much diversity in the ways and means of implementing malaria control. Over the various regions where malaria is endemic, there are numerous participants with various programmes promoting malaria control, but there is a lack of central coordination and leadership particularly in the implementation of the strategies. In the 1950s, scientific coordination was the role of the World Health Organization; now there is no real coordinating body and there are as many different strategies as there are major donors. Sadly, the problem is exacerbated by a lack of appropriately trained local scientists to design and implement strategic interventions. Moonen in the Lancet [5] has suggested that governments in endemic countries must make decisions about adopting various strategies to control or eliminate malaria, but who are the administrators to turn to for advice?

To appreciate this problem it is necessary to consider the environment within affected countries. The processes involved in malaria control require both vertical and horizontal components, all of which are part of central government. First, centrally developed and implemented, commodities that are needed must be purchased, stored and distributed appropriately to meet the national plan. These items will include insecticide-treated bed nets, insecticide for indoor residual spraying together with spraying paraphernalia, point of care diagnostic tests that are standardized and locally approved, and supplies of the locally active and approved anti-malaria drugs for distribution. Deployment of these items, maintenance of records as well as routine periodic reporting is also a central function although the provincial and district offices of the Ministry of Health are key participants. Second, there is the horizontal or community based arm to the control strategy, the public health approach. These personnel are in fact the main implementers. Their work involves the attack on the parasite, diagnosis and treatment of symptomatic cases and, essential yet difficult to implement, the process of active case detection. This phase of the intervention is designed to reduce the reservoir of the parasite population that exists in asymptomatic infections, people who are not actively ill and unlikely to seek treatment. In fact, this strategy was proposed more than 50 years ago by Yekutiel in 1960 [6] and was put into practice in Brazil in the 1990s with remarkable success [7]. However, there is no concurrence on where and when active case detection should be implemented, and the reality is that, especially in areas of seasonal transmission of malaria, the parasite reservoir remains in these asymptomatic carriers and thus mosquitoes and people will get infected and reinfected. Lack of active case detection is an important flaw in the design of any malaria control intervention, and needs to be addressed by a public health approach starting at the level of the community. To facilitate this, two important technological advances now available enable detection of at least part of this reservoir and may over time add to the armoury against the parasite. These are the point of care rapid diagnostic test for malaria, that is now used in the rural health centres, and the ubiquitous cell phone. Together, they empower the health centre staff to diagnose a malaria case and to report its presence in a time sensitive manner to the regional or district authority.

## Approach

Recently in Zambia, Kamanga *et al *[8] following incident malaria cases reported weekly by health centre attendants in the Macha area, showed that malaria disappeared from the more elevated and drier part of the countryside during the non-transmission or "dry season", but was maintained in villages around the few health centres located in a somewhat moist area of floodplain, which had a higher water table. Diagnosed cases, albeit in low numbers, were detected at various rural health centres and reported weekly, persisting through the dry part of the year. Numbers then started to increase locally for several weeks prior to expanding rapidly to the entire observation area, which included some 5,000 km^2^. Active case detection should identify the reservoir that maintains the parasites. The ebb and flow of malaria cases observed by Kamanga *et al *[8] is not unique, and it has demonstrated that case detection should be carried out during the period of low transmission. It appears that the "active" process can be targeted to some extent by the presence of local cases "passively" detected in the health centres and that by rapid reporting of cases using cell phone technology it is possible to alert the appropriate authorities to carry out targeted case detection and treatment in a timely manner. Additionally, in a follow up study in the same area, Stresman [9] showed that during the low transmission season (May-November) following diagnosed clinical cases back to their homestead lead to clusters of asymptomatic cases, some of whom were gametocyte carriers. Such clustering was not seen in simultaneous random malaria survey. There is much information to be gained from available data appropriately examined.

Designing and implementing these tactics requires a well-trained and active epidemiological service, which should be the goal of the next phase of malaria control. However, it does raise the importance of having good scientific support for the implementing Ministry of Health and this will entail developing a series of career opportunities for scientists as well as physicians within the service.

Collecting, enumerating and analysing data from a variety of rural health centres in an endemic country poses serious challenges that require specific training in epidemiology and biostatistics. There is a need for personnel to interpret and present the time sensitive data in a manner easily understandable to busy administrators. This will enable decisions to be made in a timely manner, resources mobilized and focused and the appropriate anti-malaria measures implemented. These modern technologies and the urgency to implement the intervention, require a plan to resolve the problem. There is need for a cadre of trained and proficient epidemiologists to acquire data and in a matter of a week or so, design an appropriate intervention. Additionally, over time they must be able to measure and evaluate the effect of the operation. This will necessitate training appropriate personnel. Apart from basic epidemiological principles it is necessary for the students to be exposed to special epidemiology methods designed to utilize malaria specific information and present it in a visual manner so that it can be used to communicate with decision makers, relate the alternatives available for response and then advise the field personnel of the necessary response. This expertise is available but is dependent on personnel with a good background in epidemiology. It is important that training of such specialists be carried out in country and in the local university system. This would ensure that the resources available, as well as the subject matter, are locally applicable and relevant. Facilities to transfer expertise from developed institutions to universities in endemic countries should be encouraged.

A corollary to this strategy is the creation of career opportunities for locally trained individuals, and these careers must be competitive and within the local civil service. It is important that malaria-endemic nations be encouraged to fund careers for scientists within the civil service, as this is a basis for both monitoring programmes and development of new interventions. Local scientists are a local strength and, for the overall approach to malaria control outlined in this paper to be effective, it is necessary to sustain ongoing monitoring, evaluation and implementation within the Ministry of Health. If anything was learned from the malaria situation in Zimbabwe, where this disease had been under control for nearly 50 years [10,11], it is that malaria control is totally dependent on a sustained system of surveillance, on a careful monitoring of the nature of the disease within the country, and the existence of an informed and responsive public health system.

## Competing interests

The authors declare that they have no competing interests.

## Authors' contributions

CS and PT wrote the paper based on numerous discussions, input and intellectual exchange with DS and SM. The ideas represent a joint synthesis and concern. All of us have read and approve the final manuscript.
